# Risk perception and factors associated with the acceptance of smallpox vaccine against monkeypox among healthcare and healthcare support workers in Northeastern Nigeria

**DOI:** 10.11604/pamj.supp.2025.50.1.45150

**Published:** 2025-09-25

**Authors:** Francis Enenche Ejeh, Cecelia Ballah Denue, Yusuf Madaki Lekko, Fatima Liman Shettima, Fatima Adamu Lawan, Bashir Usman Malgwi, Yasheruram Muhammad Shettima

**Affiliations:** 1 Department of Veterinary Microbiology, University of Maiduguri, Maiduguri, Nigeria,; 2 Department of Public Health, National Open University of Nigeria, Maiduguri Study Center, Maiduguri, Nigeria,; 3 Department of Geography, University of Maiduguri, Maiduguri, Nigeria,; 4 Department of Veterinary Medicine, University of Maiduguri, Maiduguri, Nigeria,; 5 Department of Veterinary Public Health and Preventive Medicine, University of Maiduguri, Maiduguri, Nigeria

**Keywords:** Emerging zoonoses, monkeypox, smallpox vaccine, vaccine acceptance, public Health

## Abstract

**Introduction:**

monkeypox, an emerging viral zoonosis, has been declared a public health emergency by the World Health Organization. The smallpox vaccine is effective for prevention against monkeypox, but the perception and acceptability of this vaccine among healthcare workers in Northeastern Nigeria remain unclear. This study aimed to evaluate risk perception and factors associated with smallpox vaccine acceptance among healthcare workers in Northeastern Nigeria.

**Methods:**

an online self-administered questionnaire was used to assess the risk perception and factors associated with smallpox vaccine acceptance among 316 healthcare and support workers in Northeastern Nigeria in a cross-sectional study. The convenience sampling method was used to recruit study participants. The researchers’ phone contacts, social media groups, and followers were invited to participate in the study. The data obtained were analyzed using SPSS version 27. Chi-square analysis determines the difference in vaccine acceptance among the dependent variables. Binary regression was employed to evaluate the relationship between the dependent variables and fixed factors. A p-value less than 0.05 was considered significant.

**Results:**

most participants (43.0%) strongly agreed that monkeypox is highly harmful, while only 9.8% believed it was less harmful than smallpox. Notably, 51% did not consider monkeypox a biological weapon aimed at reducing Africa’s population; however, 32% still held this belief. There was a significant correlation (p < 0.05) between risk perception and willingness to receive the smallpox vaccine for monkeypox, although occupational risk perception did not significantly influence vaccination willingness (p > 0.05). vaccine safety, marital status, and professions were associated with the acceptance of the smallpox vaccine.

**Conclusion:**

the healthcare workers surveyed generally felt at low risk for monkeypox yet exhibited a high willingness to accept the smallpox vaccine. Factors such as profession and perceived safety of the smallpox vaccine were linked to vaccine acceptance. Providing healthcare workers with training on vaccine safety, efficacy, and building trust regarding vaccine development is recommended.

## Introduction

Monkeypox, a zoonotic disease originating in the rainforests of Central and West Africa, is caused by the Monkeypox virus (MPXV), a member of the Orthopoxvirus family, enveloped, double-stranded DNA virus [1], which also includes smallpox. Monkeypox virus was first discovered in the late 1950s in Denmark [2,3]. The disease was first reported in the Democratic Republic of the Congo in 1970 [2-4]. Since then, there have been reports of an increase in cases observed in West Africa, including many other countries globally [5]. In 2023, 87,858 cases and 143 deaths were reported from 111 countries between January and May [3]. A majority (n = 59,413, 67.6%) of these cases were reported in the Americas, 25,902 (29.5%) in Europe, 1794 (2%) in Africa, 608 in the Western Mediterranean region, and 90 in the Eastern Pacific Region [3]. Consequently, the WHO declared Mpox a public health problem of international concern (PHEIC) [3]. There are two distinct genetic clades of MPXV1: the Congo Basin clade (now renamed as Clade I) and the West African clade (renamed as Clade II) [6].

Nigeria has reported the highest cases of Mpox in Africa as of May 2023, with 842 cases, followed by the Democratic Republic of Congo, 739, Ghana, 27, the Central African Republic, 30, and Cameroon, 29 [3]. Since the recurrence of Mpox in Nigeria from September 2017 to August 2022, a total of 985 suspected cases of Mpox have been reported, with 398 (40.4%) confirmed and 12 deaths (CFR = 3.0%) [7]. The majority of the cases were males, with 66.1%. Thirty of the 36 states and the Federal Capital Territory (FCT) have reported at least one case [7]. Most of the high-burden states in Nigeria are located within the forest belt of the country [7]. As of July 2024, Nigeria’s NCDC has reported 102 new suspected cases from eighteen States across 47 local government areas [7].

The major reason for the recent increase in Mpox cases in Nigeria is the growing contact between humans and wildlife, such as deforestation, conflict, consumption of wild animals as food, and poverty, especially in the Northeastern part of the country. Additionally, inadequate surveillance and disease monitoring systems in the country could cause cases to go unnoticed or underreported, making it challenging to track and control the outbreak [8-11]. Healthcare facilities are an important source of infectious disease transmission, and there is an estimated one healthcare worker infection per one hundred confirmed cases of Mpox. Healthcare workers are among the top priority populations for Mpox vaccination. In a study by [12], it was reported that the overall prevalence of mpox vaccine acceptance was 58.5%, and the prevalence of mpox vaccine refusal was 41.5%.

Although recent vaccines, JYNNEOS, have been reported to be associated with milder adverse events compared with previous ones, such as ACAM2000 [12], there is still a need to evaluate the perception of healthcare workers on the safety of the mpox vaccine in northeast Nigeria. Moreover, there was an earlier misinformation, particularly in northern Nigeria, in which it was said that the 2017 mpox outbreaks were due to vaccine safety. After the 1996 case of an adverse drug event that led to the death of about 11 persons, the northern part of Nigeria had been known for vaccine refusal and hesitancy [13]. Therefore, understanding the factors associated with mpox vaccine acceptance could guide the mpox prevention and control strategy in Northeastern Nigeria. Therefore, the study aimed to evaluate the risk perception and factors associated with the acceptance of the smallpox vaccine for protection against mpox among healthcare workers in northeastern Nigeria.

## Methods

**Study design:** we used a cross-sectional survey to evaluate the risk perception and acceptance of the smallpox vaccine for use against monkeypox among healthcare workers and support staff in Northeastern Nigeria. The study was conducted from March to August 2022. The methods used in this study and the questionnaire have been previously published [14] with minor modifications. The questionnaire was pre-evaluated for consistency and error following Ejeh et al. [14]. The reliability coefficient was calculated using SPSS v.20. The Cronbach´s alpha value was greater than 0.70. The data from the pilot study were not included in the final analysis.

**Study population:** participants were twenty years and above, adults, healthcare workers, and healthcare support staff residing in Northeastern Nigeria. Healthcare workers in this study include medical doctors, veterinary doctors, nurses, pharmacists, medical laboratory scientists, radiologists, dentists, public health officers, and clinical psychologists. At the same time, healthcare support staff includes veterinary and human hospital assistants such as genitors, drivers, security guards, horticulturists, engineers, mechanics, carpenters, and others. A Google Form was used to collect data from the study participants. The Google web link was mailed to the target participants. We used snowball sampling methods. This was achieved by mailing the Google link to some selected healthcare workers and healthcare support staff in the authors´ contacts (phone numbers, WhatsApp, Instagram, Telegram, Facebook, and other social media contacts). We then solicited the primary contacts to share the questionnaire web links (Google links) with their contacts and groups on social media. Reminders were sent via chart, text messages, and phone calls until the required sample size was achieved. A brief description was included in the introduction, and the submitted questionnaire was considered consent to participate in the study.

**Study setting/location:** the study was conducted in northeastern Nigeria. Northeastern Nigeria is located at a Latitude 11019´ 48 of 11.330 and a Longitude 6053´24 of 13.14. Most of the population in Northeastern Nigeria is Muslim. The region is characterized by humanitarian crises and poor healthcare facilities [14]. The study participants were from the six states in the Northeast States in Nigeria, including Borno, Yobe, Gombe, Adamawa, Taraba, and Bauchi State.

**Inclusion and exclusion criteria:** the source population included veterinarians, epidemiologists, public health officers, and other clinical staff. The clinical staff included in the study were medical doctors, nurses, pharmacists, druggists, midwives, physiotherapists, and laboratory professionals. Administrative officers, clerical staff, security, genitors, drivers, engineers, computer scientists, and others comprise the healthcare support staff. Internet and social media access, living or working in northeastern Nigeria. The subjects who were unwilling to participate and absent from the north during the data collection period were excluded.

**Measurement and description of the tool:** before developing the tool, an extensive review of the literature was done. Books and articles were also studied. Opinions and suggestions from various experts in the research were considered. The following tools were selected for data collection, which were constructed according to the study’s objective. It consists of four sections. Section A: demographic profile. Section B: risk perception, severity, and myth regarding the monkeypox disease among healthcare workers in the northeastern part of Nigeria. Section C: perception of safety of smallpox vaccine against monkeypox among healthcare workers in the northeastern part of Nigeria. Section D: factors associated with the acceptance of smallpox vaccine against monkeypox among healthcare workers in northeastern Nigeria.

**Sample size determination:** we use Ep.info TM (version 7.2.6, CDC) to calculate the minimum sample size of 174 participants to achieve our research objectives at a 95% confidence interval. Additional criteria for sample size calculation include a 13% vaccine hesitance rate in Nigeria [15], 5% bound-on-error, and a 20% non-response rate. The final sample size was 316. The sample size was increased to achieve reliable results, a better estimate, confidence, and to minimize smaller test errors. We used convenience sampling techniques.

**Statistical analysis:** the data generated was entered into Microsoft Excel and later imported into SPSS version 27 for inferential analysis. All Likert-scale responses were dichotomized for analysis. Descriptive statistics for risk perception, perception of safety of smallpox vaccine, and factors associated with smallpox vaccine acceptance were calculated and presented in tables and graphs. Differences in proportion among demographic characteristics were evaluated using Pearson´s Chi-square test or Fisher´s test as appropriate. We used the Odds ratio to evaluate the association of risk factors with smallpox vaccine acceptance among the study participants, including healthcare workers and support staff. The statistical significance level was set at p < 0.05 (two-sided).

**Ethical consideration:** the study was conducted in accordance with the principles of the Declaration of Helsinki. Participant’s confidentiality was maintained throughout the research process, and the study protocol was reviewed and approved by the ethical committee of the Faculty of Health Sciences, National Open University of Nigeria (NOUN), with the approval number (NOU214023/2021E).

## Results

D**emographic characteristics of the study participants:** three hundred and sixteen people completed the survey questionnaire in Nigeria from May to November 2022. The survey received responses from participants of diverse demographic characteristics. Table 1 showed that the study participants were mostly 20-39 years old 157 (49.7%), male 239 (75.6%), single 170 (53.8%), urban residents 252 (79.7%, university first degree 113 (35.8%), and nurses were 55 (17.4%) (Table 1).

**Table 1:** demographic characteristics of the study population

Variables	Frequency	Percentage (%)
**Age group**		
20-29	157	49.7
30-39	108	34.2
40-49	41	13.0
50 and above	10	3.2
**Gender**		
Male	239	75.6
Female	77	24.4
**Marital status**		
Single	170	53.8
Married	146	46.2
**Residential location**		
Urban	252	79.7
Rural	64	20.3
**Education**		
Secondary	63	19.9
Diploma/NCE/HND	76	24.1
First degree	113	35.8
Postgraduate	53	16.8
Primary	11	3.5
**Profession**		
Medical doctor	40	12.7
Nurse	55	17.4
Veterinary doctor	29	9.2
Public health officer	16	5.1
Laboratory scientist	22	7.0
student	24	7.6
Pharmacist	41	13.0
Others	89	28.2
**Total**	**316**	**100.0**

**Figure 1:**
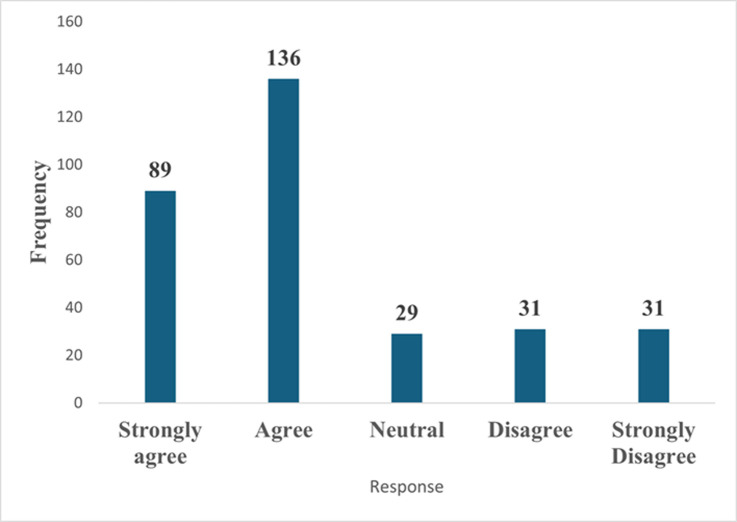
monkeypox is like a smallpox disease and is generally very harmful

**Figure 2:**
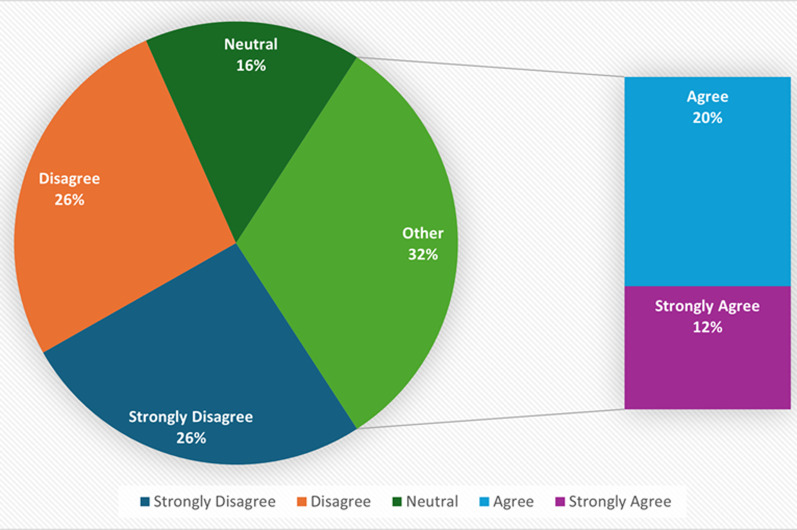
monkeypox is a biological weapon for reducing the African population

**Risk perception, severity, and myth regarding monkeypox disease among healthcare workers in the northeastern part of Nigeria:** concerning risk perception, 100% of the study respondents believe that they were not at risk of monkeypox because of occupational exposure. Figure 1 compares the responses of the respondents to whether monkeypox is as severe as smallpox. The majority, 136 (43.0%), strongly agreed that monkeypox was very harmful, while only 31 (9.8%) held that monkeypox was not as harmful as smallpox. Figure 2 showed that the majority (51%) of the respondents believed that monkeypox was not a biological weapon for reducing the population of Africans. Sixteen percent (16%) were undecided, while 32% still held that monkeypox was a biological weapon.

**Figure 3:**
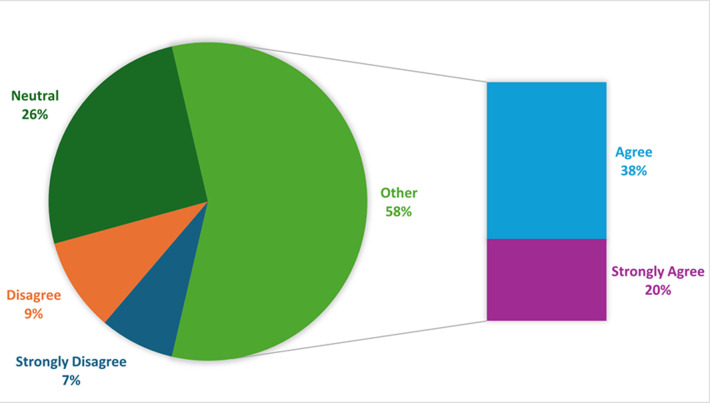
the use of the smallpox vaccine against monkeypox is safe

**Figure 4:**
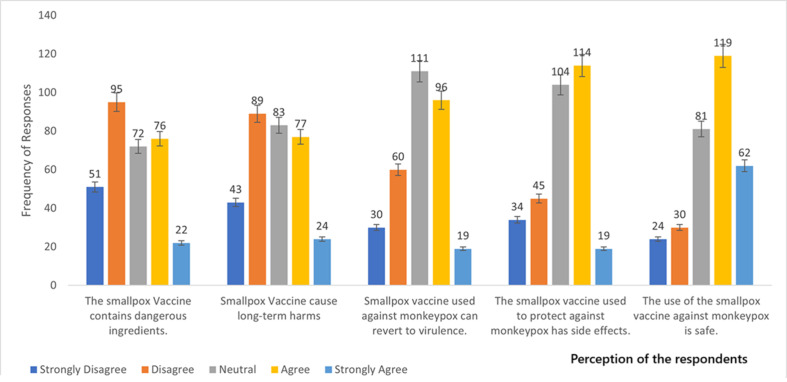
perception of the respondents about the safety of the smallpox vaccine

**Perception of safety of smallpox vaccine against monkeypox among healthcare workers in the northeastern part of Nigeria:** more than half (58%) of the respondents believed that the smallpox vaccine was safe for use against monkeypox. However, about 18% still held that the smallpox vaccine was not safe. While 26% were undecided (Figure 3). Although an aggregate of 133 (42.1%) agreed that the smallpox vaccine has side effects, 79 (25.0%) disagreed, while 104 (32.9%) remained neutral. When asked if the smallpox vaccine can revert to virulence, only 90 (28.5%) disagreed, and 111 (35.1%) were neutral. Generally, the perception of the respondents concerning the safety of the smallpox vaccine against monkeypox was not favorable (Figure 4). Regarding the demographic characteristics and perception of safety of smallpox vaccine among healthcare and healthcare support workers in northeast Nigeria. We observed that the perception of safety of smallpox vaccine differs significantly (χ2 = 9.962; p-value = 0.019) among the different age groups. The age group 20-29 had 63 (40.1%) followed by 40-49 years old 13 (31.7%). Marital status differs significantly (χ2 = 12.333; p-value = 0.000). The single had 70 (41.2%) while the married had 33 (22.6%). Surprisingly, participants with the lowest level of education had the highest (81.8%) perception rate (Table 2).

**Table 2:** demographic characteristics and perception of smallpox vaccine safety among healthcare workers in northeastern Nigeria

Variables	Negative perception (%)	Positive perception (%)	Chi-square (χ^2^)	P-value
**Age group**				
20-29	94 (59.9)	63 (40.1)	9.962	0.019
30-39	82 (75.9)	26 (24.1)		
40-49	28 (68.3)	13 (31.7)		
50 and above	9 (90.0)	1 (10.0)		
**Gender**				
Male	155 (64.9)	84 (35.1)	2.906	0.088
Female	58 (75.3)	19 (24.7)		
**Marital status**				
Single	100 (58.8)	70 (41.2)	12.333	0.000
Married	113 (77.4)	33 (22.6)		
**Location**				
Urban	170 (67.5)	82 (32.5)	0.002	0.967
Rural	43 (67.2)	21 (32.8)		
**Education**				
Primary	2 (18.2)	9 (81.8)		
Secondary	30 (47.6)	33 (52.4)	45.110	0.000
Diploma	44 (57.9)	32 (42.1)		
First degree	90 (79.6)	23 (20.4)		
Postgraduate	47 (88.7)	6 (11.3)		
**Occupation**				
Healthcare workers	130 (66.7)	65 (33.3)	0.126	0.722
Non-healthcare workers	83 (68.6)	38 (31.4)		
**Profession**				
Medical doctors	24 (60.0)	16 (40.0)		
Nurses	33 (60.0)	22 (40.0)	17.584	0.014
Veterinary doctors	25 (86.2)	4 (13.8)		
Public health officer	13 (81.2)	3 (18.8)		
Laboratory scientists	18 (81.8)	4 (18.2)		
Students	14 (58.3)	10 (41.7)		
Pharmacists	21 (51.2)	20 (48.8)		
Others	65 (73.0)	24 (27.0)		
**Acceptance of Smallpox Vaccine**				
Yes	148	73	0.064	0.801
No	65	30		
Total	213 (67.4)	103 (32.6)		

**Factors associated with the acceptance of the smallpox vaccine against monkeypox among healthcare workers and healthcare support staff in northeastern Nigeria:** about 221 (70.0%) of the respondents were willing to accept the smallpox vaccine. The willingness to accept the smallpox vaccine was not significantly (p< 0.05) different among the demographic variables. But the age group 30-39 had the highest percentage, 77 (71.3%), of the intention to vaccinate against Mpox by using the smallpox vaccine, followed by 50 years and above, 7 (70.0%). Male participants had 174 (72.8%) and females had 47 (61.0%) smallpox vaccine acceptance rates. Rural residents had a higher 45 (70.3%) smallpox vaccine intention than urban dwellers 176 (69.8%). Healthcare workers who had postgraduate education had a higher rate of 41 (77.4%) smallpox vaccine intention than those with lower academic qualifications. Also, public health officers 13 (81.2%) had the highest acceptance rate, followed by pharmacists 31 (75.6%) (Table 3).

**Table 3:** demographic characteristics and the willingness to accept the smallpox vaccine among healthcare workers in Nigeria

Variables	Intent to receive	Percentage (%)	Chi-square (p-value)
**Age group**			
20-29	109	69.4	0.167 (0.983)
30-39	77	71.3	
40-49	28	68.3	
50 and above	7	70.0	
**Gender**			
Male	174	72.8	3.834 (0.050)
Female	47	61.0	
**Marital status**			
Single	112	65.9	2.877 (0.090)
Married	109	74.7	
**Residential location**			
Urban	176	69.8	0.005 (0.941)
Rural	45	70.3	
**Education**			
Primary	7	63.6	5.489 (0.241)
Secondary	47	74.6	
Diploma/NCE/HND	46	60.5	
First Degree	80	70.8	
Postgraduate	41	77.4	
**Profession**			
Medical doctor	29	72.5	7.972 (0.335
Nurse	32	58.2	
Veterinary doctor	21	72.4	
Public Health Officer	13	81.2	
Laboratory Scientist	14	63.6	
student	20	83.3	
Pharmacist	31	75.6	
Others	61	68.5	

Risk perception varies significantly (p < 0.05) regarding the willingness to vaccinate against Mpox by using smallpox (Table 4). Perception of occupation risk did not differ significantly (p>0.05) with the willingness to vaccinate against Mpox. Respondents who identified that they were occupationally exposed had 98 (72.6%), while those who were not occupationally exposed had 83 (67.5%) smallpox acceptance rates. Respondents who strongly agreed 53, 85.5%) that the smallpox vaccine is safe had the highest acceptance rate, while those who disagreed that the smallpox vaccine was safe had 14 (46.7%) (Table 4). Multivariable regression analysis revealed that respondents who agreed that the smallpox vaccine is safe were about three times (AOR: 3.13; 95% CI: 1.90-5.15; p = 0.000) more likely to accept the smallpox vaccine against monkeypox than those who did not agree. Surprisingly, respondents who agreed that the smallpox vaccine contained harmful ingredients were 3.01 times (AOR: 3.01; 95% CI: 1.84-4.92; p = 0.000) more likely to accept the vaccine. Also, students and pharmacists were more likely to accept the smallpox vaccine for protection against Mpox (Table 5).

**Table 4:** risk perception and the willingness to accept the smallpox vaccine among healthcare workers in Northeastern Nigeria

High risk because of occupational exposure	Intent to receive (%)	Chi-square	P-value
Disagree	98 (72.6)	0.832	0.660
Neutral	40 (69.0)		
Agree	83 (67.5)		
**Monkeypox is fatal**			
Strongly disagree	23 (74.2)	1.868	0.760
Disagree	23 (74.2)		
Neutral	20 (69.0)		
Agree	90 (66.2)		
Strongly agree	65 (73.2)		
**Smallpox vaccination is safe**			
Strongly disagree	18 (75.0)	**27.619**	**0.000**
Disagree	14 (46.7)		
Neutral	44 (54.3)		
Agree	92 (77.3)		
Strongly agree	53 (85.5)		
**A means to reduce the African population**			
Strongly disagree	67 (81.7)	**13.466**	**0.009**
Disagree	60 (71.4)		
Neutral	26 (52.0)		
Agree	42 (66.7)		
Strongly agree	26 (70.3)		
**Smallpox vaccine causes long-term harm**			
Strongly disagree	36 (83.7)	9.317	0.054
Disagree	67 (75.3)		
Neutral	50 (60.2)		
Agree	51 (66.2)		
Strongly agree	17 (70.8)		
**The smallpox vaccine contains harmful ingredients**			
Strongly disagree	39 (76.5)	**17.243**	**0.002**
Disagree	74 (77.9)		
Neutral	40 (55.6)		
Agree	48 (63.2)		
Strongly agree	20 (90.9)		
**Smallpox vaccine can revert to virulence**			
Strongly disagree	28 (93.3)	**11.451**	**0.022**
Disagree	43 (71.7)		
Neutral	75 (67.6)		
Agree	60 (62.5)		
Strongly agree	15 (78.9)		
**The smallpox vaccine has side effects**			
Strongly disagree	26 (76.5)	5.081	0.279
Disagree	36 (80.0)		
Neutral	66 (63.5)		
Agree	79 (69.3)		
Strongly agree	14 (73.7)		

**Table 5:** factors associated with willingness to accept the smallpox vaccine for the prevention of Mpox among healthcare workers

Variables	COR	95% CI	P-value	AOR	95% CI	P-value
**Age group**	1.70	0.29-9.89	0.555			
20-29	1.05	0.20-5.66	0.953			
30-39	0.88	0.15-5.10	0.887			
40-49	1	1	1			
50 and above						
**Gender**						
Male	1.90	0.97-3.73	0.062	1.71	0.99-2.93	0.052
Female	1	1	1			
**Marital status**						
Single	0.31	0.15-0.65	0.002	0.65	0.40-1.07	0.091
Married	1	1	1			
**Residential location**						
Urban	1.23	0.57-2.66	0.600			
Rural	1	1	1			
**Education**						
Secondary	2.01	0.36-11.10	0.422			
Diploma/NCE/HND	2.06	0.35-12.32	0.426			
First Degree	2.39	0.39-14.59	0.345			
Postgraduate	2.43	0.19-1.01	0.380			
Primary	1	1	1			
**Profession**						
Medical doctor	3.24	0.97-10.83	0.056	1.21	0.53-2.76	0.651
Nurse	1.27	0.46-3.45	0.642	0.64	0.32-1.28	0.208
Veterinary doctor	2.39	0.69-8.26	0.168	1.21	0.48-3.05	0.694
Public Health Officer	5.24	0.93-29.73	0.061	1.99	0.55-7.54	0.312
Laboratory Scientist	1.30	0.38-4.53	0.684	0.80	0.30-2.13	0.660
student	9.92	2.05-47.91	0.004	2.30	0.71-7.34	0.161
Pharmacist	3.86	1.23-12.06	0.020	1.14	0.61-3.30	0.411
Others	1	1	1			
**High risk because of occupational exposure**						
Agree	0.55	0.29-1.06	0.076	0.83	0.51-1.35	0.447
Disagree	1	1	1			
**Monkeypox is fatal**						
Agree	0.60	0.31-1.18	0.140			
Disagree	1	1	1			
**Smallpox vaccination is safe**						
Agree	9.09	4.47-18.46	0.000	3.13	1.90-5.15	0.000
Disagree	1	1	1	1	1	1
A means to reduce the African population						
Agree	0.68	0.27-1.90	0.413			
Disagree	1	1	1			
**The smallpox vaccine causes long-term harm**						
Agree	0.41	0.15-1.14	0.087	0.84	0.50-1.40	0.488
Disagree	1	1	1	1	1	1
**The smallpox vaccine contains harmful ingredients**						
Agree	3.07	1.09-8.61	0.033	3.01	1.84-4.92	0.000
Disagree	1	1	1	1	1	1
Smallpox vaccine can revert to virulence						
Agree	0.33	0.15-0.76	0.007	0.71	0.43-1.16	0.167
Disagree	1	1	1	1	1	1
**The smallpox vaccine has side effects**						
Agree	0.98	0.48-1.99	0.953	0.99	0.61-1.63	0.997
Disagree	1	1	1	1	1	1
						

COR: cude odds ratio, CI: confidence interval, AOR: adjusted odds ratio

## Discussion

This study is important because we are in an era when infectious disease frontliners are constantly exposed to the risk of acquiring emerging and re-emerging diseases of animal origin referred to as zoonoses, and which we know very little about [16]. Therefore, efforts must be put in place to prevent the spread of these diseases, like Mpox, among healthcare workers and healthcare facilities. One of the ways by which Mpox can be prevented from spreading in health facilities and the community is by acquiring immunity through vaccination [17]. This study evaluated the factors that are associated with the acceptance of the smallpox vaccine against Mpox among healthcare workers in Nigeria. And outline areas that should be addressed to prevent the spread of Mpox among healthcare workers.

Although Mpox is not yet as severe as smallpox, our findings revealed that most of the respondents had a low perception of the seriousness of Mpox. The perception of the seriousness of an infectious disease has been recognized as a factor that was driving the COVID-19 vaccine acceptance rate [18,19]. Mpox is an evolving viral zoonosis, and the biology, transmission, virulence, and pathophysiology of the disease are not yet fully understood [16]. Hence, the fear of the unknown is an intrinsic factor that may enhance adherence to preventive measures such as vaccination against Mpox.

Another important finding of this study regarding misinformation was the question of whether Mpox is a biological weapon for targeting the African population. Most of the respondents agreed that Mpox was not a biological weapon. But 32% agreed that Mpox was a weapon to reduce the population in Africa. This finding was worrisome because healthcare workers in this study were expected to have accurate knowledge about Mpox. Since they are regarded in the community as leaders and bearers of accurate health information. Also, the communities in Nigeria rely on the healthcare workers for authentic health information. It is recommended that healthcare workers be made to participate in continuing professional education with a focus on emerging and re-emerging zoonoses and one health. The reason for this finding may be because of the proliferation of myths and misinformation aided by social media. Social media has been reported as the major driver of infodemics during the COVID-19 pandemic. Infodemics could have a negative effect on vaccine acceptance. Therefore, healthcare workers are recommended to seek health information from verifiable sources such as professional colleagues, government websites, conferences, workshops, symposiums, and international organizations such as the World Health Organization (WHO), and the World Organization for Animal Health (WOAH).

We observed that many of the participants agreed that the use of the smallpox vaccine to prevent Mpox is safe. Although a large proportion (26.0%) of the respondents were neutral, about 16% were worried that the smallpox vaccine was not safe for use against monkeypox. This finding is worrisome. But our result was consistent with a recent study in China in which the authors reported that a large (60.7%) proportion of respondents were worried about the adverse effect of the vaccine [20]. The reason for this finding may be attributed to misinformation and poor knowledge about Mpox.

Furthermore, most of the healthcare workers in this study agreed that the smallpox vaccine had side effects (42.1%) and could revert to virulence (36.4%). Also, 213 (67.4%) of the healthcare workers in this study had a negative perception of smallpox vaccine safety. The ripple effect of the recent COVID-19 pandemic´s myths and misinformation spread by social media [15] may be responsible for the high negative perception about the use of the smallpox vaccine against monkeypox among HCWs in northeastern Nigeria. Also, northern people of Nigeria, particularly, the Northwest, have had some negative experience with the introduction of new vaccine and drug trials [21,22]. One of the benefits of vaccination is national health security, with the attendant reduction in poverty. Although northeastern Nigeria has the highest poverty rate, high illiteracy, and the poorest health indicators in Nigeria. The northern region, including the northeast, witnessed a major vaccination boycott in 2003 [22] because of a general distrust [23] and the fear that the vaccine was contaminated with harmful antifertility and sterilizing agents [21]. Another reason for the high negative perception may be that the smallpox vaccine is new to many people in the present generation. The perception of smallpox vaccine safety among age groups varies significantly (χ2 = 9.962, p = 0.019). Healthcare workers (HCWs) who were 20-29 years old had the highest positive perception of smallpox vaccine safety compared with other age groups. Also, HCWs who were single had significantly higher positive perception about the smallpox vaccine than those who were married. The reason for this finding may be that most of the single HCWs were within the age group 20-29.

Healthcare workers that had primary and secondary education demonstrated higher positive perception toward smallpox vaccine safety than those with higher education, this finding is consisted of [24-26]. Among the health professionals, medical doctors (40.0%), nurses (40.0%), students (41.7%) and pharmacologist (48.8%) had high proportion of HCWs that expressed position perception of safety towards smallpox vaccine. This observation was not surprising because the core healthcare workers had longer years of training, more conferences and workshops, and continued professional education. We recommend that local or regional trust networks led by healthcare workers and other stakeholders, like the traditional and religious leaders, can be drivers of the vaccination campaign. Therefore, policymakers, government agencies, and non-governmental organizations could build trust within the networks to boost vaccine acceptance in the community.

Although smallpox vaccination is not available in Nigeria because of the global eradication of smallpox [27], our findings revealed that 221 (70;0%) of the HCWs intended to receive the smallpox vaccine for protection against Mpox whenever it is available. This agrees with previous studies [26,28]. The proportion of those who decline to vaccinate against Mpox by using the smallpox vaccine was low (30%), but this is of public health concern, given that healthcare workers are regarded as ambassadors or vaccine crusaders. Besides, HCWs who are at high occupational risk were recommended to vaccinate against monkeypox by using the smallpox vaccine [29,30].

The overall willingness and refusal to vaccinate against Mpox by using the smallpox vaccine were found to be 221 (70.0%) and 95 (30.0%), respectively, among healthcare workers in Northeastern Nigeria. Although we found no significant (p > 0.05) between vaccine acceptance and demographic variables, risk perception differs significantly with the acceptance of the smallpox vaccine against Mpox. Respondents who perceived that the smallpox vaccine was safe had a higher acceptance rate than those who did not. Also, we observed that the perception that the smallpox vaccine was safe was about three times more likely to accept vaccination. This finding was no surprise since safety is one of the critical characteristics of vaccines [24,31,32]. Vaccination campaign effects should ensure that the public, and particularly healthcare workers, receive adequate information on vaccine safety to enhance vaccination compliance.

Furthermore, vaccine manufacturers must ensure that vaccines produced for human use meet the highest safety standards because our findings showed that respondents who perceived that the smallpox vaccine may contain a harmful ingredient were less likely to receive the vaccine. In northeastern Nigeria, safety issues and the perception that the previous vaccine contains antifertility and harmful ingredients have had a negative impact on vaccine acceptance [21-23]. The vaccine acceptance reported in this study was higher than a previous meta-analysis to examine the acceptance of the monkeypox vaccine globally [33,34] reported that the overall vaccine acceptance rate was 64% among physicians, 84.0% among LGBTI, and 56.0% among the general public. In another study [20], reported a lower vaccine acceptance among healthcare workers. The vaccine acceptance rate in this study was found to be lower than that of a study conducted in Indonesia [35,36]. The difference in the acceptance rate may be due to differences in cultural, economic, and demographic characteristics of the study participants.

Limitations of the study: for starters, our research is limited to northeast Nigeria. Thus, the study´s findings may not represent the willingness to accept the smallpox vaccine for protection against Mpox throughout the country, as there have been more cases of monkeypox in the south than in the north. Another limitation of this study was that the respondents were selected from among the contacts of the authors and their associates. Hence, the information provided in this study does not represent the general opinion of healthcare workers in the region. However, the study had generated baseline research data that will be useful for future research and as a guide for future health policies regarding Mpox prevention and control in the region.

## Conclusion

Our findings revealed that most of the healthcare workers in this study perceived that Mpox was very harmful. But three out of every ten healthcare workers in northeastern Nigeria who participated in the study refused to accept vaccination against Mpox. Even though most of the respondents agreed that the smallpox vaccine was safe for use against Mpox. Being a student and pharmacist were found to be more likely to accept Mpox vaccination, while the perception that the smallpox vaccine could revert to virulence was less likely to accept vaccination against Mpox by using the smallpox vaccine. Safety is a critical issue regarding vaccine production and vaccination campaigns. Therefore, be guided jealously.

### What this study adds

- Monkeypox can affect both young and adults;
- There is cross-protection of the smallpox vaccine against mpox;
- The Mpox vaccination acceptance rate differs among different populations.

### What is known about this topic

- The current study reported low perception of the seriousness of mpox and low perception of the safety of the smallpox vaccine among healthcare workers and support staff in northeast Nigeria;
- Despite the low perception of safety of the smallpox vaccine among healthcare workers and support staff in this study, most of the participants were willing to receive the smallpox vaccine;
- The study further highlights the factors that may hinder smallpox vaccine acceptance among healthcare workers and support staff in northeast Nigeria.
